# Integrated Characterization of Cassava (*Manihot esculenta*) Pectin Methylesterase (*MePME*) Genes to Filter Candidate Gene Responses to Multiple Abiotic Stresses

**DOI:** 10.3390/plants12132529

**Published:** 2023-07-03

**Authors:** Shijia Wang, Ruimei Li, Yangjiao Zhou, Alisdair R. Fernie, Zhongping Ding, Qin Zhou, Yannian Che, Yuan Yao, Jiao Liu, Yajie Wang, Xinwen Hu, Jianchun Guo

**Affiliations:** 1 College of Life Sciences, Hainan University, Haikou 570228, Chinaliruimei@itbb.org.cn (R.L.);; 2 Institute of Tropical Bioscience and Biotechnology, Chinese Academy of Tropical Agricultural Sciences, Haikou 571101, China; 3 Max Planck Institute of Molecular Plant Physiology, 14476 Potsdam-Golm, Germany; fernie@mpimp-golm.mpg.de; 4 College of Chemical and Materials Engineering, Hainan Vocational University of Science and Technology, Haikou 571126, China

**Keywords:** pectin methylesterase, cassava, postharvest physiological deterioration, gene family, abiotic stress

## Abstract

Plant pectin methylesterases (PMEs) play crucial roles in regulating cell wall modification and response to various stresses. Members of the PME family have been found in several crops, but there is a lack of research into their presence in cassava (*Manihot esculent*), which is an important crop for world food security. In this research, 89 *MePME* genes were identified in cassava that were separated into two types (type-Ⅰ and type-Ⅱ) according to the existence or absence of a pro-region (PMEI domain). The *MePME* gene members were unevenly located on 17 chromosomes, with 19 gene pairs being identified that most likely arose via duplication events. The *MePMEs* could be divided into ten sub-groups in type-Ⅰ and five sub-groups in type-Ⅱ. The motif analysis revealed 11 conserved motifs in type-Ⅰ and 8 in type-Ⅱ *MePMEs*. The number of introns in the CDS region of type-Ⅰ *MePMEs* ranged between one and two, and the number of introns in type-Ⅱ *MePMEs* ranged between one and nine. There were 21 type-Ⅰ and 31 type-Ⅱ *MePMEs* that contained signal peptides. Most of the type-Ⅰ *MePMEs* had two conserved “RK/RLL” and one “FPSWVS” domain between the pro-region and the PME domain. Multiple stress-, hormone- and tissue-specific-related *cis*-acting regulatory elements were identified in the promoter regions of *MePME* genes. A total of five co-expressed genes (*MePME1*, *MePME2*, *MePME27*, *MePME65* and *MePME82*) were filtered from different abiotic stresses via the use of UpSet Venn diagrams. The gene expression pattern analysis revealed that the expression of *MePME1* was positively correlated with the degree of cassava postharvest physiological deterioration (PPD). The expression of this gene was also significantly upregulated by 7% PEG and 14 °C low-temperature stress, but slightly downregulated by ABA treatment. The tissue-specific expression analysis revealed that *MePME1* and *MePME65* generally displayed higher expression levels in most tissues than the other co-expressed genes. In this study, we obtain an in-depth understanding of the cassava *PME* gene family, suggesting that *MePME1* could be a candidate gene associated with multiple abiotic tolerance.

## 1. Introduction

Pectin methylesterases (PMEs) are one kind of cell-wall-associated enzyme that have the ability to render the pectin demethylesterified [1]. The degree of methylesterification of pectin determines its functional properties, and affects the cell wall firmness, thereby regulating the plant cellular response to different vital activities, such as growth, development, senescence and response to (a)biotic stresses [2,3]. PME activity is, therefore, frequently measured as a proxy in evaluating plant resistance to different stress conditions. For example, PME activity in chilling-sensitive maize was reduced using low temperature treatment with the pectin content in the cell wall being simultaneously decreased [4]. During strawberry fruit ripening, PME activity also gradually decreased [5]. By contrast, during tomato fruit ripening, PME activity gradually increased [6]. On the exogenous application of PME combined with calcium chloride, the fruit softening of raspberry was delayed due to an extended maintenance of cell wall integrity [7]. Moreover, by increasing the PME activity, the phosphorus fixed in the cell wall was remobilized [8]. During lettuce seed germination, the PME activity was lower in the endosperm cap, but higher in the radicle [9]. When suffering aluminum (Al) stress, the PME activity in the root tip of an Al-sensitive rice variety was maintained at a higher level than that in a resistant variety [10]. PME activity is also induced when the plant suffers a pathogen attack [11]. In all cases, PME enzymatic activity is tightly regulated by PME inhibitor proteins (PMEIs) [3,12,13].

In seed plants, *PMEs* belong to a super-family that is present at different numbers in different species. According to existing reports, the number of PME family members in different plant species ranges from 41 in foxtail millet [14] to 121 in tobacco [15]. In addition, the number of *PME* genes reported in dicotyledonous plants is generally higher than that reported in monocotyledonous plants. For example, in the monocots rice [16] and foxtail millet [14], the *PME* numbers are under 50, whilst in the dicot plants potato [17], *Arabidopsis* [18], *Populus euphratica*, tobacco [15], flax [19], navel orange [20], tomato [21] and *Brassica rapa* [22], the numbers of *PME* are 54, 66, 80, 121, 105, 53, 79 and 110, respectively. The PMEs initially encode pro-proteins with pro-regions or signal peptides, which are crucial for protein orientation in the endoplasmic reticulum. The pro-proteins undergo a series of processing in the Golgi and are finally secreted to the cell wall in a mature state (with pro-region removed). According to the reported *PME* gene families of different plant species, the number of type-Ⅰ *PMEs* (with pro-region) is usually larger than that of type-Ⅱ *PMEs* (without pro-region). For example, there are 28 type-Ⅰ and 26 type-Ⅱ *PMEs* in potato, 43 type-Ⅰ and 23 type-Ⅱ *PMEs* in *Arabidopsis*, 36 type-Ⅰ and 35 type-Ⅱ *PMEs* in peach and 47 type-Ⅰ and 33 type-Ⅱ *PMEs* in cotton [17,18,23,24].

Some reports have shown that the expression of *PME* genes is affected by plant growth and development and stress. For example, *AtPME41* in *Arabidopsis* [25] and nine *CsPME* genes in navel orange were induced by low temperature treatment [20]. In contrast, *AtPME34* was induced by heat stress and ABA; additionally, it was highly expressed in guard cells [26], whilst *AtPME2* was associated with lateral root emergence and hypocotyl elongation, stomatal development and guard cell movement [27,28]. Moreover, *AtPME35* was involved in regulating stem mechanical strength [29], *AtPME6* was increased during embryo development [30] and *AtPME31* was induced by salt stress [31]. Similarly, *PME* genes in rice, rye and tea were induced by aluminum stress [10,32,33,34]. Furthermore, *PME* gene expression was dynamically regulated during fruit ripening and postharvest storage [23,35,36,37].

Cassava (*Manihot esculenta*) is a species of the Euphorbiaceae, with considerable agronomic value. The cassava storage roots are full of starch, which can be used not only for food, but also for ethanol production and medicine [38,39,40]. Cassava has several advantages as a crop staple such as its high tolerance to heat, drought and poor soil, but it also has some disadvantages such as a susceptibility to mosaic virus infection, the tendency to easily suffer postharvest deterioration as well as an intolerance to salt and low temperatures [41,42]. In recent years, the function of several genes in cassava have been analyzed [43,44,45,46]. However, the function of *PME* family gene members in this species remains unclear. To rectify this, in the current study, the *PME* family genes of cassava were systematically analyzed, and key multiple functional genes were identified. As such, this in-depth study lays a theoretical foundation for further functional validation of the utility of *MePME* genes.

## 2. Results

### 2.1. The Manihot Esculenta Genome Contains 89 PME Genes

We identified 89 *MePME* genes in the cassava genome (V6.1) using the keyword search for the PFAM domain Pectinesterase (Pfam01095). According to whether there is a pro-region PMEI (Pfam04043) within the amino acid sequence, these PMEs were separated into two sub-types, i.e., type-Ⅰ PMEs with the PMEI domain and type-Ⅱ PMEs without the PMEI domain. In this study, we found 44 type-Ⅰ *MePMEs* and 45 type-Ⅱ *MePMEs* in cassava (Table S1).

**Figure 1:**
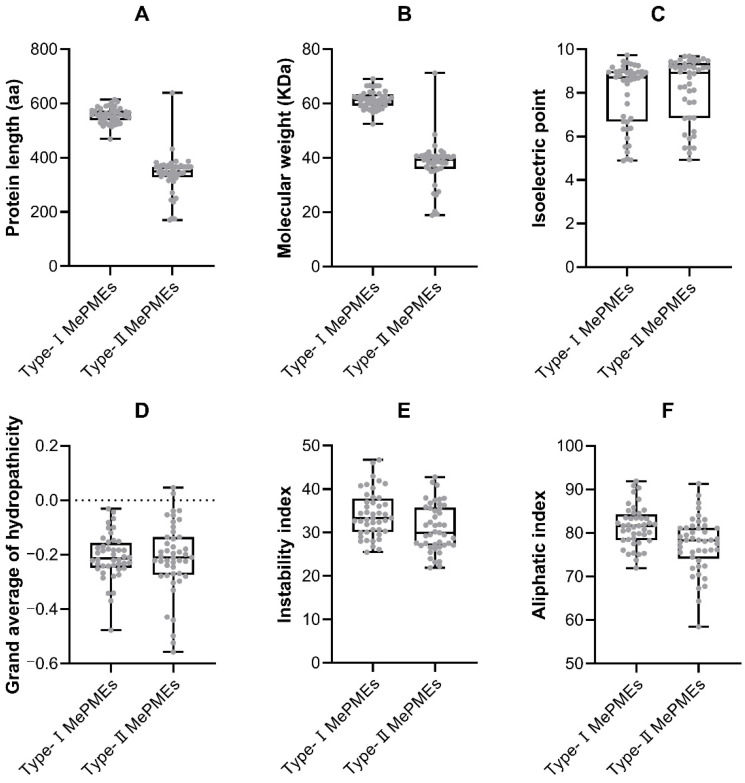
Physical and chemical parameters analysis of two types of *MePMEs* in cassava genome v6.1. (**A**) Protein length analysis of *MePMEs*; aa: amino acid. (**B**) Molecular weight analysis of *MePMEs*; KDa: kilo Dalton. (**C**) Isoelectric point analysis of *MePMEs*. (**D**) Protein hydrophobic analysis of *MePMEs*. (**E**) Protein stability analysis of *MePMEs*. (**F**) Protein aliphatic index analysis of *MePMEs*.

Various physical and chemical parameters of *MePMEs* were assessed using the ProtParam algorithm embedded in the Expasy website [47], revealing that the protein length of type-Ⅰ *MePMEs* ranged from 469 aa (*MePME*38) to 614 aa (*MePME*28), with most of them being around 550 aa, while the protein length of type-Ⅱ *MePMEs* ranged from 170 aa (*MePME*43) to 639 aa (*MePME*37), with most of them being around 350 aa (Table S1, Figure 1A). The molecular weights of type-Ⅰ *MePMEs* ranged from 52.49 KDa (*MePME*38) to 69.11 KDa (*MePME*28), while those of type-Ⅱ *MePMEs* ranged from 18.89 KDa (*MePME*24) to 71.25 KDa (*MePME*37) (Table S1, Figure 1B). The isoelectric points (pIs) of type-Ⅰ *MePMEs* ranged from 4.89 (*MePME*16) to 9.74 (*MePME*44), while those of type-Ⅱ *MePMEs* ranged from 4.94 (*MePME*12) to 9.69 (*MePME*7). Most of the *MePMEs* (30 out of 44 in type-Ⅰ, and 33 out of 45 in type-Ⅱ) are alkaline proteins, as their pI values are greater than 7, whilst the rest of the *MePMEs* (14 in type-Ⅰ, 12 in type-Ⅱ) are acidic proteins (Table S1, Figure 1C). All type-Ⅰ *MePMEs* are hydrophilic proteins due to their grand average hydrophobic indices being below zero. Most type-Ⅱ *MePMEs* (43 out of 45) are also hydrophilic proteins, except for *MePME*24 and *MePME*89 (Table S1, Figure 1D). The protein stability analysis indicated that most *MePMEs* (37 out of 44 in type-Ⅰ, and 42 out of 45 in type-Ⅱ) are instable proteins exhibiting instability indices lower than 40 (Table S1, Figure 1E). The protein aliphatic indices of type-Ⅰ *MePMEs* ranged from 71.92 (*MePME*68) to 91.91 (*MePME*78), while those of type-Ⅱ *MePMEs* ranged from 58.47 (*MePME*18) to 91.32 (*MePME*89) (Table S1, Figure 1F).

### 2.2. Chromosome Distribution and Collinear Analysis of MePMEs

**Figure 2:**
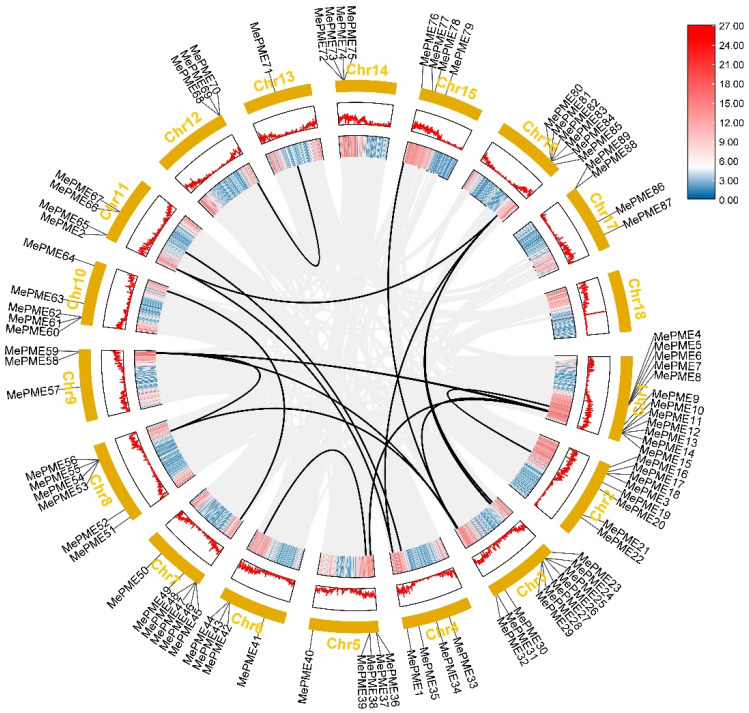
Distribution and collinearity analysis of pectin methylesterase family genes on chromosomes in cassava genome v6.1. Chr is the abbreviation of chromosome; the black lines indicate pairwise replication of *MePMEs*.

The chromosome localization analysis indicated that 87 of the *MePMEs* were randomly located across the 17 chromosomes, whereas the chromosome positions of two *MePMEs* (*MePME88* and *MePME89*) could not be determined (defined as ChrS) (Table S1, Figure 2). In addition, chromosome 1 (Chr01) contains the largest number of *MePME* genes, with a total of twelve (three type-Ⅰ, and nine type-Ⅱ). Chr03 has ten *MePMEs*, including six type-Ⅰ and four type-Ⅱ *MePMEs*. Chr02 has eight *MePMEs*, including two type-Ⅰ and six type-Ⅱ *MePMEs*. Chr07, Chr08 and Chr16 all have six *MePMEs*, with five type-Ⅰ and one type-Ⅱ *MePMEs*. Chr05 and Chr10 both have five *MePMEs* including two type-Ⅰ and three type-Ⅱ *MePMEs*. Chr04, Chr06, Chr11, Chr14 and Chr15 all have four *MePMEs*, of which Chr04, Chr11 and Chr15 contain two type-Ⅰ and two type-Ⅱ *MePMEs*, while Chr06 contains one type-Ⅰ and three type-Ⅱ *MePMEs*, and Chr14 only contains four type-Ⅱ *MePMEs.* Chr09 and Chr12 both have three *MePMEs,* of which Chr09 has one type-Ⅰ and two type-Ⅱ *MePMEs,* while Chr12 only has three type-Ⅰ *MePMEs*. Chr17 has two type-Ⅰ *MePMEs* and ChrS has two type-Ⅱ *MePMEs.* Chr13 has one gene, *MePME71,* which is a type-Ⅰ *MePME*. The collinearity analysis revealed that 19 *MePME* pairs likely resulted from gene duplication events in cassava (Figure 2, Table S2). There are thirteen type-Ⅰ *MePME* pairs, six type-Ⅱ *MePME* pairs and no gene duplication events leading to *MePMEs* pairs of different types. In addition, *MePME* duplication occurred only between different chromosomes, but not on the same chromosome. It was noted that there were duplication relationships among the three type-Ⅰ genes, *MePME1*, *MePME2* and *MePME80*. The type-Ⅱ *MePME32* had duplication relationships with three other type-Ⅱ genes, namely, *MePME13*, *MePME59* and *MePME76*.

### 2.3. Phylogenetic Analysis of MePMEs

**Figure 3:**
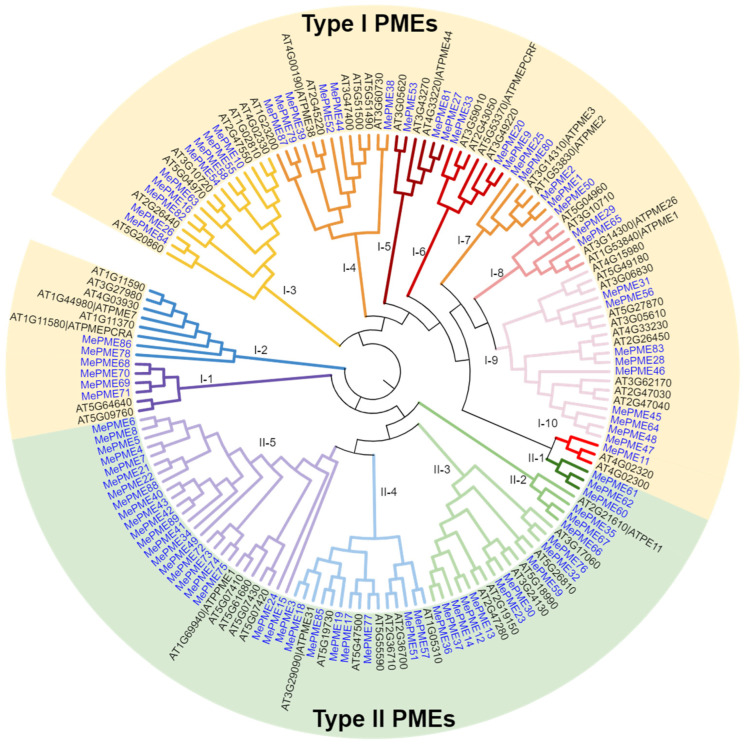
Phylogenetic trees of PME family proteins in cassava genome v6.1 and *Arabidopsis genome TAIR10.* The phylogenetic relationship was calculated based on maximum likelihood method and Jones–Taylor–Thornton model and was visualized using iTOL v6. The yellow background indicates type-Ⅰ PMEs, and the green background indicates type-Ⅱ PMEs.

To better understand the relationship between PMEs, a phylogenetic tree was drawn linking 89 cassava PMEs and 66 *Arabidopsis* PMEs following a multiple sequence alignment via ClustalW, tree construction via the neighbor-joining method using MEGA 11 software [48] and graphic beautification using iTOL [49]. As presented in Figure 3, we were able to divide type-Ⅰ PMEs into ten sub-groups (Ⅰ-1~10), and type-Ⅱ PMEs into five sub-groups (Ⅱ-1~5). Most groups had roughly similar numbers of genes in both cassava and *Arabidopsis*. However, in group Ⅰ-2, two cassava *MePMEs* clustered with six *Arabidopsis* PMEs. In group Ⅱ-5, 21 *MePMEs* clustered with 5 PMEs from *Arabidopsis*. It is worth noting that *Arabioposis* AtPME2 and AtPME3, which have been reported to have stress-resistance functions, clustered with four *MePMEs* (including *MePME*1, *MePME*2, *MePME*25 and *MePME*80) in group Ⅰ-7. *Arabidopsis* AtPME31, which has been characterized to play a role in salt tolerance, clustered with cassava *MePME*3 and *MePME*18 in group Ⅱ-4.

### 2.4. Gene Structure and Motif Analysis of MePME Genes in Cassava

**Figure 4:**
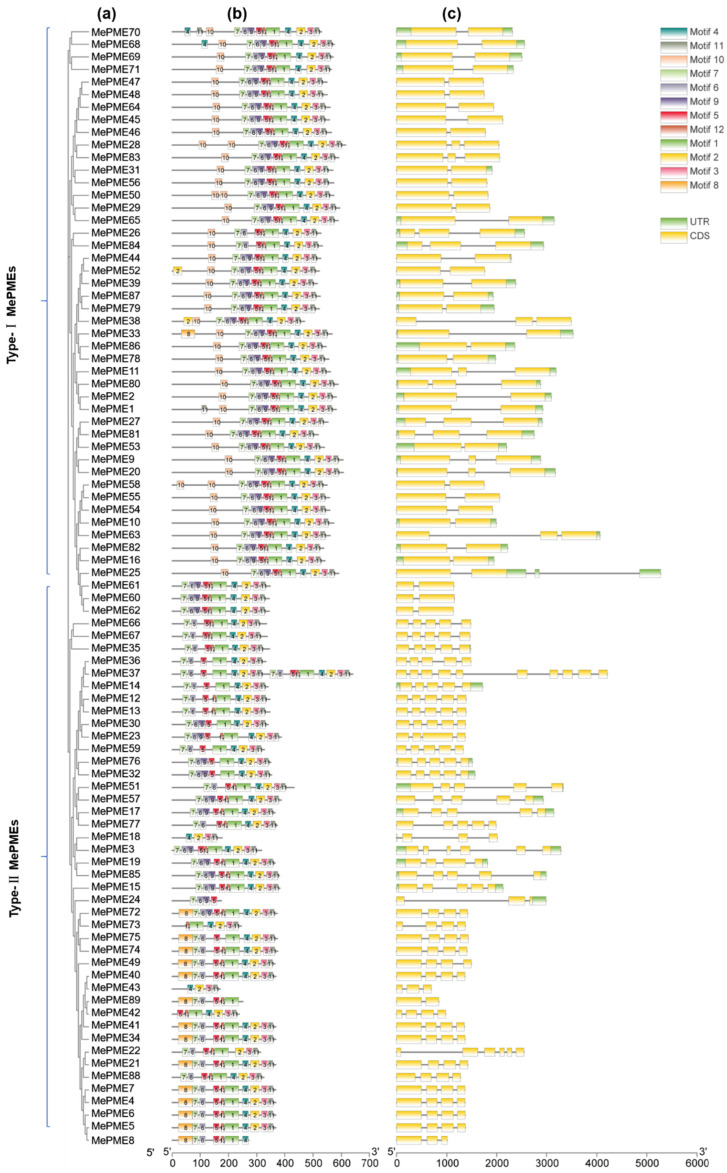
Integrated analysis of phylogenetic relationship (**a**), motifs (**b**) and structure (**c**) of *MePME* family members in cassava genome v6.1.

The amino acid sequences of 89 *MePMEs* were used to draw a phylogenetic tree, using the MEGA11 software, in which the two types of *MePMEs* were well separated (Figure 4a). Twelve kinds of motifs were searched for within the *MePMEs*’ amino acid sequences using MEME analysis (Figure 4b). Each type-Ⅰ *MePME* contains 11 motifs including motifs 1~7 and motifs 9~12, indicating that these 11 motifs are conserved in type-Ⅰ *MePMEs*. In type-Ⅱ *MePMEs*, motifs 1~7 and motif 11 are conserved, as they appear in each type-Ⅱ *MePME* (Figure 4b). In addition, motif 8 was only apparent in one of the type-Ⅰ *MePMEs*, namely, *MePME*33, while it appeared in 13 type-Ⅱ *MePMEs* (Figure 4b). The gene structure analysis revealed that most type-Ⅰ *MePMEs* (32 out of 44) have only one intron within their CDS, while 12 of them have two introns. In addition, 25 type-Ⅰ *MePMEs* have both up- and down-stream UTRs, while four genes have only down-stream UTRs (Figure 4c). The intron numbers in type-Ⅱ *MePMEs* varied from one to nine. Moreover, eleven type-Ⅱ *MePMEs* had up- or down-stream UTRs (Figure 4c). By and large, the more analogous the *MePME* genes, the more similar the motifs and gene structures (Figure 4).

### 2.5. Domain Analysis of MePMEs

**Figure 5:**
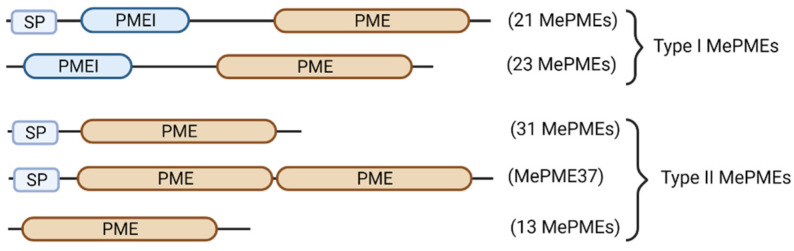
Different domain distribution patterns of *MePMEs* in cassava genome v6.1. SP, signal peptide. This figure was created using BioRender.com.

**Figure 6:**
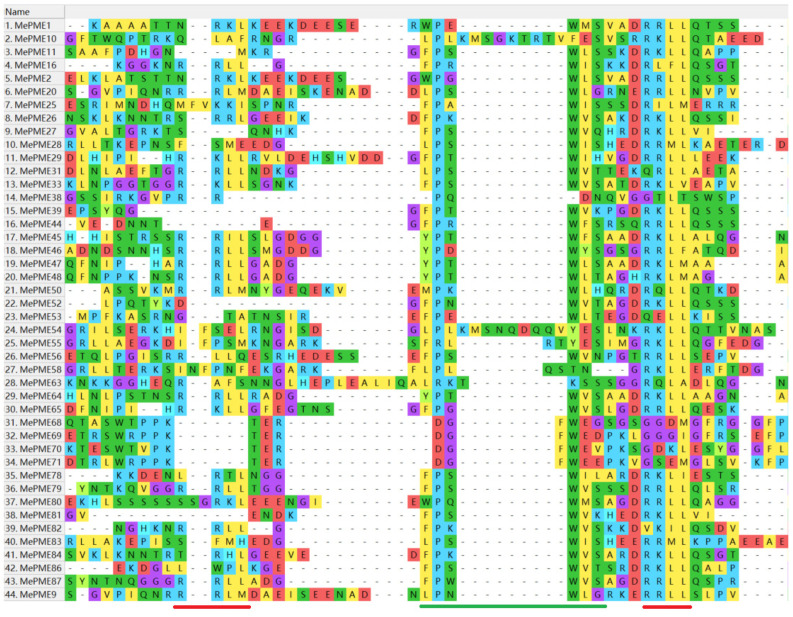
Conserved basic motifs in type-Ⅰ *MePMEs* from cassava genome v6.1. Red line indicates the “RK/RLL” basic motif, and green line indicates the “FPSWVS” motif.

To survey the domains in cassava *MePMEs*, the 89 *MePME* amino acid sequences were analyzed. Each of the 44 type-Ⅰ *MePMEs* contains one PMEI domain and one PME domain. Most (44 out of 45) type-Ⅱ *MePMEs* contain only one PME domain, except *MePME*37, which has two PME domains (Figure 5). In 52 out of 89 *MePMEs* (21 type-Ⅰ and 31 type-Ⅱ), a signal peptide was predicted with high confidence by using the SignalP program (Table S3). Through multiple sequence alignment, we found that most type-Ⅰ *MePMEs* contain two basic RK/RLL motifs (Figure 6), with the second one being more conserved than the first one. In addition, between the RK/RLL motifs, there is usually an “FPSWVS” motif; here, in type-Ⅰ *MePMEs*, the amino acid residues “P” and “W” are relatively more conserved. Only in four type-Ⅰ *MePMEs* (*MePME*68~71), the “P” was replaced with “D”, whilst the “W” in six type-Ⅰ *MePMEs* including *MePME*10, *MePME*38, *MePME*54, *MePME*55, *MePME*58 and *MePME*63 was changed into “F”, “-”, “Y”, “Y”, “T” and “K”, respectively (Figure 6). These changes may affect the function of *MePMEs*.

### 2.6. Cis-Acting Regulatory Elements Analysis of MePMEs

**Figure 7:**
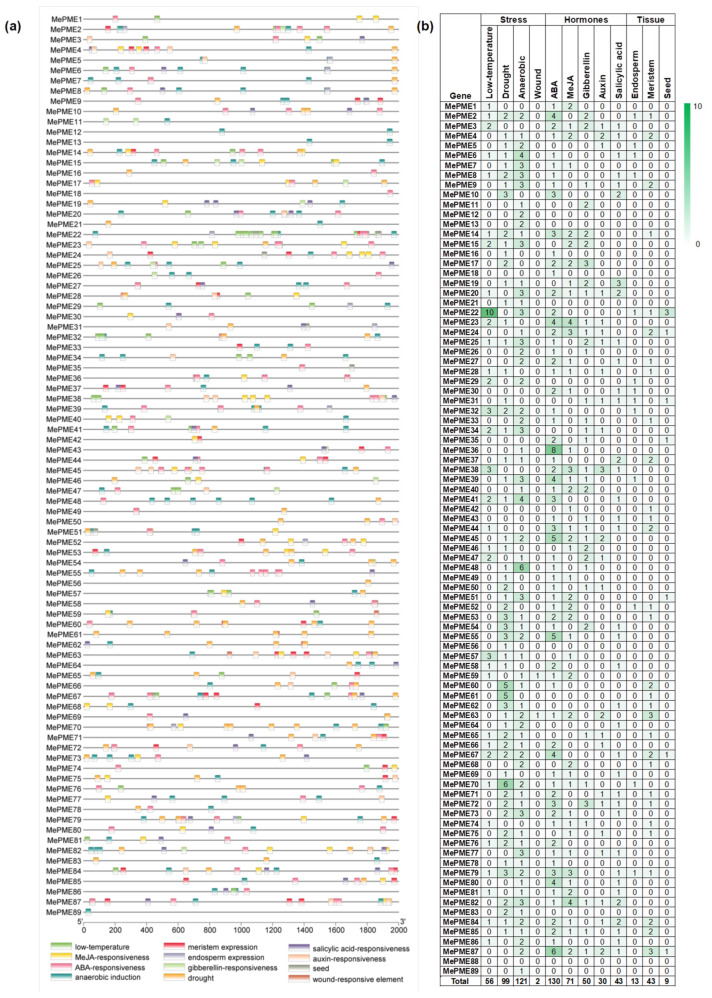
Important *Cis*-acting regulatory element localization (**a**) and number analysis (**b**) in *MePME* promoters from cassava genome v6.1.

Cis-acting regulatory elements (CAREs) are important binding sites on the promoter region of genes for regulating gene expression. We predicted stress-, hormone- and tissue-specific-related CAREs on the *MePMEs* promoters (2000 bp upstream of initiation codon “ATG”). The results display that the types and quantities of CAREs in the promoter regions of *MePMEs* were diverse (Figure 7). The vast majority of *MePMEs* members possessed at least one type of stress-related and hormone-related CARE. In detail, 33 *MePMEs* had at least one low-temperature-related CARE, 57 *MePMEs* had drought-response-related CAREs, and 64 *MePMEs* had anaerobic-induction-related CAREs. Only two *MePMEs* (*MePME59* and *MePME63*) harbored wound-response CAREs. For hormone-related CAREs, ABA-response-related CAREs appeared in 66 *MePMEs*, MeJA-response-related CAREs appeared in 41 *MePME*s, gibberellin-response-related CAREs existed in 30 *MePMEs*, auxin-response-related CAREs were found in 24 *MePMEs* and salicylic-acid-response-related CAREs appeared in 36 *MePMEs*. These results indicate that *MePMEs* play roles in response to stress and are regulated by hormones in cassava. In addition, some *MePMEs* harbor tissue-specific expression-related CAREs, such as endosperm, meristem and seed. This may relate to the special function of these tissues.

### 2.7. Expression Analysis of MePMEs in Cassava under Different Stresses

**Figure 8:**
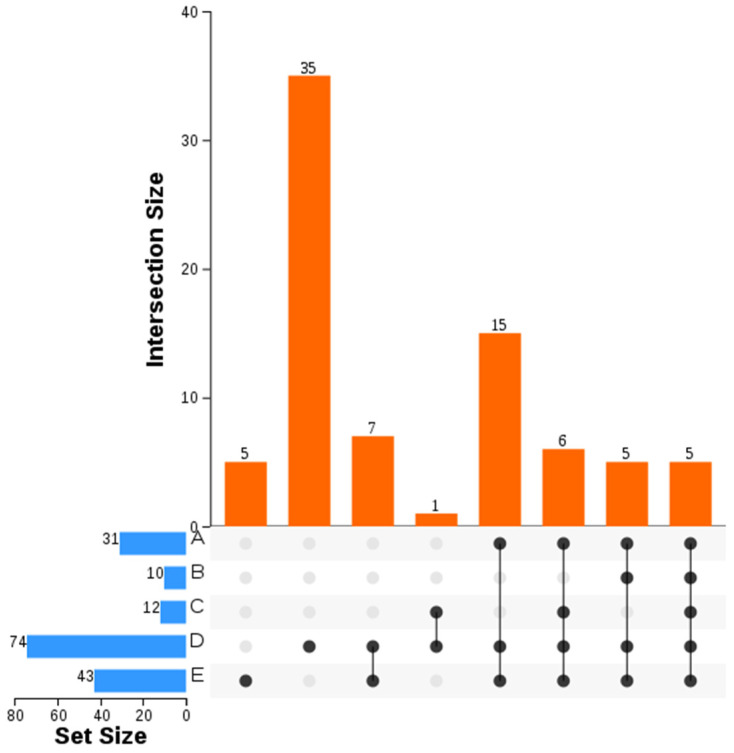
UpSet Venn diagram illustrating shared genes in different transcriptome data of cassava. A: *MePME* genes appear with FPKM values in each sample in transcriptome data of PPD-tolerant cassava (RYG1) and PPD-sensitive cassava (SC8) following storage for 0 and 21 days. B: *MePMEs* appear in transcriptome data of SC124 cassava at 0, 6, 12 and 48 h after section, and are significantly differentially expressed in at least one comparison after filter via fold change ≥ 2 and *p*-value ≤ 0.05. C: *MePMEs* appear in transcriptome data of melatonin- and water (control)-treated cassava at 6, 12 and 48 h after section, and are significantly differentially expressed in at least one comparison between melatonin and control after filter via fold change ≥ 2 and *p*-value ≤ 0.05. D: *MePMEs* appear with FPKM values in each sample in transcriptome data of cassava treated with different chilling stress. E: *MePMEs* appear with FPKM values in each sample in transcriptome data of cassava treated with 50 μmol/L ABA and 7% PEG for 48 h, separately. The blue horizontal bar chart on the left shows the element statistics for each collection. A single black dot in the middle matrix represents an element specific to a certain set. The lines between points represent the unique intersection of different sets. The red vertical bar chart represents the corresponding intersection element values, respectively.

Postharvest physiological deterioration (PPD) rapidly occurs after cassava is harvested, and severely restricts the economic value of the crop. A previous study indicated that PME activity and expression was associated with fruit postharvest quality. Therefore, we analyzed the expression of *MePMEs* in cassava transcriptome data in response to PPD. A total of 31 *MePME* genes with values of transcription per kilobase per million mapping readings (FPKM) in each sample were found in the transcriptomic data of two cassava varieties (PPD-sensitive cassava SC8 and PPD-tolerant cassava RYG1) after 0- and 21-day storage (SC8 severely deteriorated, while RYG1 maintained good quality) [41]. A total of ten *MePME* genes were found to participate in the response to PPD in the transcriptome data of SC124 cassava during PPD at time points of 0 h (control), 6 h (no symptoms of PPD), 12 h (local symptoms of PPD) and 48 h (global PPD symptoms) after deterioration [50]. A total of twelve *MePMEs* were reported to be associated with melatonin-delayed PPD in SC124 [51] (Table S3). As a tropical crop, cassava is sensitive to low temperatures. To reveal the response mechanism of cassava to chilling stress, a transcriptomic analysis of a mixture of leaf and root samples was performed for plants that were kept at 24 °C (CK), at 14 °C for 5 days (T1), at 14 °C for 5 days followed by 4 °C for 5 days (T2) and at 24 °C for 5 days followed by 4 °C for 5 days (T3) [52]. We found that a total of 73 *MePMEs* had FPKM data in each sample (Table S3). Cassava has a high tolerance to drought. ABA acts as a crucial signal in response to drought stress. To reveal whether *MePMEs* are responsive to drought stress and the ABA signal, the transcriptome data of cassava treated with 7% polyethylene glycol (PEG) and 50 μmol/L abscisic acid (ABA) were analyzed. A total of 43 *MePMEs* had FPKM data in both the PEG- and ABA-treated cassava (Table S3). To select crucial *MePME* genes displaying multiple stress responses, UpSet Venn diagrams were used to filter the *MePMEs* shared by PPD, chilling stress, ABA and PEG treatments. The results show that there are five *MePME* genes in all of the above transcriptomes—namely, *MePME1*, *MePME2*, *MePME27*, *MePME65* and *MePME82* (Figure 8, Table S3). Interestingly, all five shared genes are type-Ⅰ *MePMEs*.

**Figure 9:**
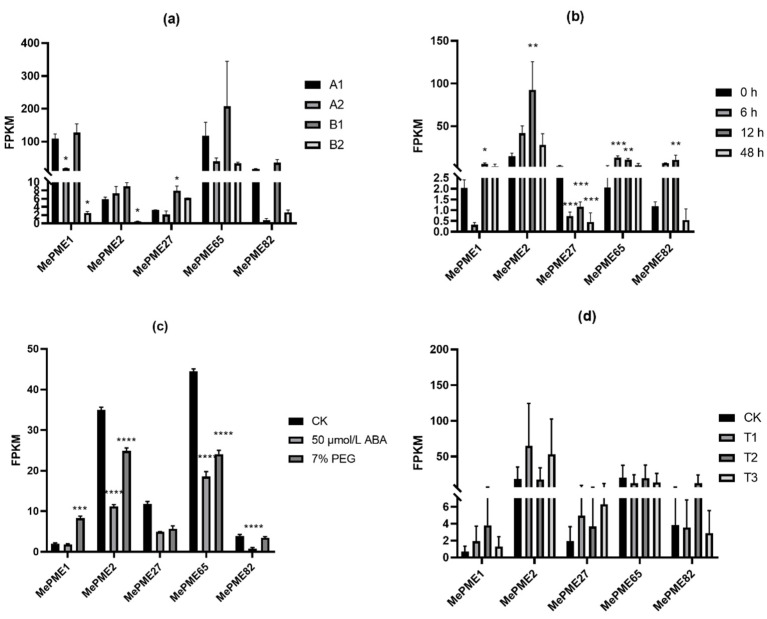
Expression patterns of five shared *MePMEs* under different stresses in cassava. (**a**) Expression patterns of five shared *MePMEs* after storage in two cassava varieties with different PPD tolerance. A1: 0 days of PPD-sensitive cassava SC8; A2: 21 days of storage of SC8; B1: 0 days of PPD-tolerant cassava RYG1; B2: 21 days of storage of RYG1. (**b**) Gene expression patterns of five shared *MePMEs* during SC124 cassava PPD occurrence 0, 6, 12 and 48 h after section. (**c**) Gene expression patterns of five shared *MePMEs* in response to 50 μmol/L ABA and 7% PEG stresses. (**d**) Gene expression patterns of five shared *MePMEs* in response to different chilling treatments. CK: control (24 °C, 10 days); T1: chilling acclimation (24 °C, 5 days + 14 °C, 5 days); T2: chilling after chilling acclimation (14 °C for 5 days followed by 4 °C for 5 days); T3: chilling shock (24 °C for 5 days followed by 4 °C for 5 days). Values are means and standard deviations. The ordinary one-way ANOVA analysis and Dunnett’s multiple comparisons test were used to analyze the significant difference. * indicates the significant difference *p* ≤ 0.05, ** indicates the significant difference *p* ≤ 0.01, *** indicates the significant difference *p* ≤ 0.001, **** indicates the significant difference *p* ≤ 0.0001.

The expression patterns of the five shared *MePMEs* in response to PPD (in different PPD-tolerant cassava and in early stage of PPD occurrence), chilling, ABA and PEG stresses were analyzed. The expression levels of all the five shared *MePMEs* were a bit higher at t0 in RYG1 than in SC8, but these differences were not significant. After 21 days of storage, the expression levels of most of the shared *MePMEs* were inhibited in both SC8 and RYG1. Particularly, the downregulation of *MePME1* expression in RYG1 was even greater than that in SC8 (Figure 9a). During the early stages of PPD, when no obvious PPD symptoms appeared in the slices, the expressions of *MePME*1 and *MePME27* were inhibited, whereas the expression of *MePME2*, *MePME65* and *MePME82* were increased. When the PPD symptoms appeared in some of the slices 12 h after sectioning, the expressions of *MePME1*, *MePME2*, *MePME65* and *MePME82* were significantly upregulated, whereas the expression of *MePME27* was significantly downregulated. When the PPD symptoms appeared in all surfaces of slices at 48 h after sectioning, the expressions of *MePME1*, *MePME*2 and *MePME*65 were still higher than those in the control, but less than those at 12 h, whereas the expressions of *MePME*27 and *MePME*82 at 48 h were decreased compared to those in the control (Figure 9b). The expression level of *MePME1* was significantly induced by 7% PEG-simulated drought stress, while the expression of four other genes, *MePME2*, *MePME27*, *MePME65* and *MePME82,* were inhibited (Figure 9c). The expressions of all five shared *MePMEs* were decreased to various degrees after 50 μmol/L ABA treatment; in particular, the expressions of *MePME2*, *MePME65* and *MePME82* were significantly inhibited (Figure 9c). The expression levels of *MePME1*, *MePME2* and *MePME27* were slightly upregulated after T1 and T3 treatments, while the expressions of *MePME65* and *MePME82* were slightly inhibited. In addition, the expressions of *MePME1* and *MePME82* were increased after T2 treatment (Figure 9d). In general, *MePME1* showed a relatively strong response to the above adversity treatments.

### 2.8. Expression of Shared MePMEs in Different Tissues of Cassava

**Figure 10:**
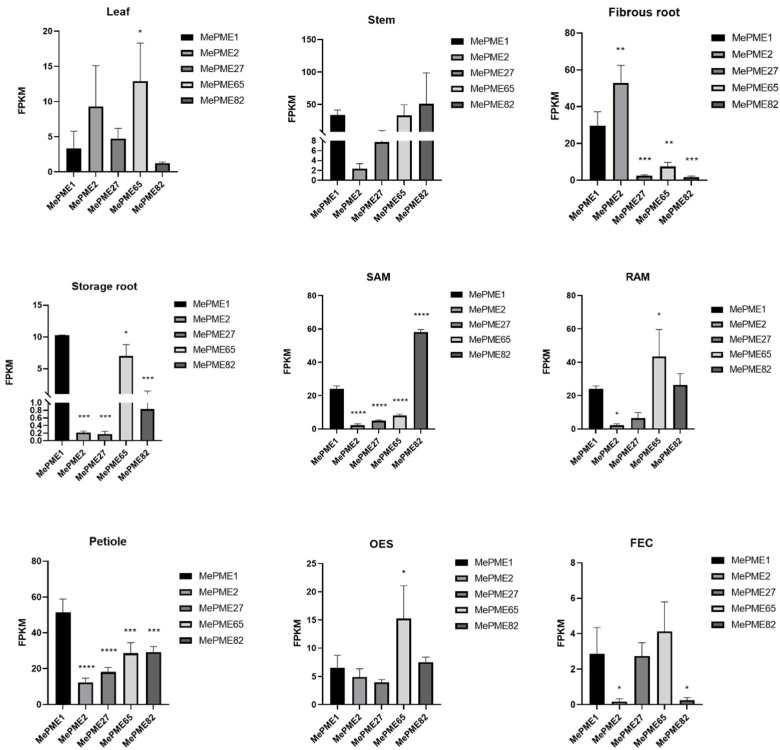
Expression pattens of co-expression *MePMEs* in different tissues of cassava. SAM, stem apex meristem. RAM, root apex meristem. OES, organized embryonic structure. FEC, friable embryogenic callus. Values are means and standard deviations. The ordinary one-way ANOVA analysis and Dunnett’s multiple comparisons test were used to analyze the significant difference. * indicates the significant difference *p* ≤ 0.05, ** indicates the significant difference *p* ≤ 0.01, *** indicates the significant difference *p* ≤ 0.001, **** indicates the significant difference *p* ≤ 0.0001.

The expression of genes in certain tissues may be related to the differentiation and development of these tissues or the special functions performed by these tissues. To know the tissue-specific expression patterns of the five shared *MePME* genes, we compared their expression levels in nine different tissue types including leaf, stem, fibrous and storage root, stem and root apex meristem (SAM and RAM), petiole, organized embryonic structure (OES) and friable embryogenic callus (FEC) (Figure 10). Overall, the five shared *MePMEs* were expressed to varying degrees in the nine tissues. An analysis of the top two genes that were highly expressed in each tissue showed that *MePME65* and *MePME2* are the top two genes in leaves, *MePME82* and *MePME1* are the most expressed in stems, while *MePME2* and *MePME1* predominate in fibrous roots, and *MePME1* and *MePME65* are mostly expressed in storage roots. Meanwhile, *MePME82* and *MePME1* are the most expressed in SAM, and *MePME65* and *MePME82* are the most expressed in RAM, whilst *MePME1* and *MePME65* predominate in petiole, *MePME65* and *MePME82* predominate in OES and *MePME65* and *MePME1* predominate in FEC. In addition, the above analysis results also show that both *MePME1* and *MePME65* were ranked in the top two for a total of six times. These results indicate that *MePME1* and *MePME65* may play a critical role in these tissues.

## 3. Discussion

The pectin methylesterase family genes have attracted considerable research attention due to their vital role in the regulation of cell wall modification. The systematic analysis of PME family genes was achieved in *Arabidopsis* [18] and several other plant species, including rice [16], tomato [21] and foxtail millet [14]. However, the abundance and function of *PME* genes in other plant species remain unclear. Herein, 89 cassava *PME* genes were uncovered and subjected to bioinformatic analysis at the whole-genome level.

Compared to the reported *PME* families in other dicots, the *PME* gene family in cassava is of mid size. However, compared to the monocots, such as foxtail millet (forty-one members) [14] and rice (forty-four members) [16], there are nearly twice as many *PMEs* in cassava. The difference in the gene family size among different species is most likely due to genome-wide duplication events. According to our research, 19 paralogous pairs (13 pairs in type-Ⅰ, and 6 pairs in type-Ⅱ) were present among the 89 *MePME* genes (Figure 2). Duplication events were also present in other species. For example, in tobacco, nine duplication pairs were identified [15]. In citrus, 23 pairs of *PME* genes were identified as duplicated genes [20]. In *Brassica rapa*, twelve *PME* gene pairs were found to have experienced duplication [22].

By comparing the PME sequences between cassava and *Arabidopsis*, the cassava PME were distinguished and further sub-divided into ten and five sub-groups (Figure 3). It is worth noting that *MePME1*, *MePME2* and *MePME80* are duplicates of each other (Figure 2). The phylogenetic relationship between them is closest and they additionally exhibit a higher similarity with AtPME2 and AtPME3 from *Arabidopsis* in group Ⅰ-7 (Figure 3). This indicates that they may have a similar biological function as AtPME2 and AtPME3.

According to previous studies, type-Ⅰ PMEs contain two conserved “RK/RLL” domains and one “FPSWVS” domain between the pro-region and PME domain. These domains were considered to be important for the cleavage of the pro-region to generate mature PME proteins [53]. In our study, most *MePMEs* contain two conserved “RK/RLL”, but some do not contain this domain (Figure 6). For the “FPSWVS” domain in cassava, “P” and “W” were more conserved than the other amino acid residues (Figure 6). However, whether the missing cleavage domains or the changed amino acid residues affect the pro-region cleavage remains unclear, and experimental evidence will likely be required in order to address this.

*Cis*-acting regulatory elements (CAREs) are important DNA sequences in the promoters of genes, which could bind to corresponding transcription factors in order to regulate gene transcription under different conditions. In this study, the CAREs in the promoter region of *MePME* family members were analyzed. We found a great deal of CAREs that respond to hormones such as abscisic acid, methyl jasmonate, auxin, gibberellin and salicylic acid. We additionally found some CAREs that respond to stresses including low-temperature, drought and anaerobic and wounding stresses. Finally, we found a set of CAREs associated with tissue-specific expression in the endosperm, meristem and seed (Figure 7). Similar findings were reported for the tobacco *PME* family members, where low-temperature-, wound-, anaerobic-, ABA-, auxin- and MeJA-related CAREs exist [15]. These results indicate that *MePMEs* may respond to a variety of abiotic stresses and participate in plant growth and development. A few studies confirmed that some transcription factors regulate the expression of *PME*. For instance, the transcription factor BELLRINGER (BLR) is a key transcription factor that regulates *AtPME5*-mediated organ initiation and cell extension [54]. The transcription factor *CsRVE1* was confirmed to directly bind to the promoter of *CsPME3* in navel orange [20]. Two BEL1-like homeodomains (BLHs) *BLH2* and *BLH4* bind to the promoter of *PME58* to activate its expression in seed mucilage [55]. *MYB80* was confirmed to target one *PME* gene in *Arabidopsis* [56]. In strawberry, the *FvMYB79* can activate the expression of *FvPME38* and lead to ABA-dependent fruit softening after harvest [57]. However, the transcription factors regulating PME expression in cassava remain to be studied.

Some previous studies proved the functions of several *PME* genes in regulating abiotic stresses. For instance, *AtPME41* was induced by chilling stress in *Arabidopsis* [25]. Nine *CsPME* genes were upregulated after low temperature treatment for 6 h [20]. Similarly, *AtPME34* was associated with heat stress, with the *pme34* mutant being sensitive to heat stress [26]. In addition, *AtPME34* was highly expressed in guard cells and was induced by ABA [26]. The expression of *AtPME2* was improved during lateral root emergence and hypocotyl elongation [27]. Furthermore, both *AtPME2* and *AtPME3* participated in stomatal development, and regulated guard cell movement [28]. *AtPME35* was specifically expressed in *Arabidopsis* in the basal part of the inflorescence stem, and was involved in regulating the stem’s mechanical strength [29]. The expression of *AtPME6* was increased during mucilage secretion and played an important role in regulating embryo development [30]. Meanwhile, *AtPME31* was induced by salt stress, and when it was knocked out, the plants showed hypersensitivity to salt stress [31], whereas in apple, the expression profile of an *AtPME2*-similar gene *MdPME2* was downregulated during apple mealiness development [35]. Similarly, in rice, six *OsPME* genes were induced by aluminum stress, the overexpression of *OsPME14* decreased the resistance of transgenic plants to aluminum stress [10,32] and three peach *PME* genes were highly expressed in melting flesh during peach fruit ripening [23]. By contrast, one *PME* gene in rye was inhibited by aluminum stress in tolerant cultivar [33]. In tea plants, 8 *PME* genes in leaves and 15 genes in roots responded to aluminum stress [34]. These studies thus highlight a common mechanism by which plants respond to different stresses, namely, the regulation of cell wall modifications involving PME, which is often overlooked, because it was previously thought that the cell wall was a relatively stable cell structure that mainly played a role in maintaining cell morphology and plant rigidity. On the further development of research in the area, the cell wall was found to be highly dynamic with the growth of the plant and changes in response to the external environment [58,59,60,61]. As one of the main components of the cell wall, pectin not only plays a crucial role in cell wall remodeling, but also participates in intercellular communication, morphogenesis and environmental sensing [60]. PME is the first enzyme in the process of pectin degradation, which regulates the homeostasis of pectin in the cell wall, and thus, mediates the remodeling of the cell wall in response to changes in the external environment [62].

As an important food and renewable bioenergy raw material that is widely cultivated in tropical and subtropical regions of the world, cassava has many advantages and disadvantages. It is very suitable for arid and barren soil, but it is very sensitive to low temperatures, and its storage roots very readily deteriorate after harvest [41,63]. In this study, we filtered out five *MePMEs* (*MePME1*, *MePME2*, *MePME27*, *MePME65* and *MePME82*), which were shared by different stress treatments, namely, PPD, chilling, 50 μmol/L ABA and 7% PEG stresses. Although studies on the function of the PME gene are still relatively few, some studies have shown that a single *PME* gene has multiple functions. *AtPME3* was rapidly induced by *Pectobacterium carotovorum* and *Botrytis cinerea,* and caused sensitivity to Zn^2+^ following mis-expression [64,65]. *AtPME31* could improve the resistance of transgenic plants to salt stress and various insect pests [31,66]. *AtPME34* was induced by ABA and participated in stomatal movement and heat stress response [26]. In tobacco, *NtPME043* was induced by both salt and ABA stress [15]. It is speculated that these genes may be involved in cell wall modification under various stresses. Additionally, among the shared *MePMEs*, we found that the expression of *MePME1* was significantly induced during the occurrence of PPD 12 h after the cassava tuberous roots were sliced. In PPD-resistant cassava RYG1, in which no PPD symptoms occurred after 21 days of storage, the expression of *MePME1* was significantly downregulated (Figure 9). These results indicate that *MePME1* plays a crucial role in regulating cassava resistance to PPD. The expression of *MePME1* was also increased following chilling acclimation (14 °C for 5 days), and by using 7% PEG treatment (Figure 9). Notably, *MePME1* was also the most highly expressed of the five selected genes in storage root and petiole. Moreover, it was also the second highest expressed of these genes in the stem, fibrous root, shoot apex meristem, and friable embryogenic callus (Figure 10). These results suggest that *MePME1* should be subjected to further investigation.

In summary, eighty-nine PME genes were identified in cassava. An integrated characterization of the *MePMEs* in cassava was performed through the analysis of their physical and chemical properties, chromosome localization and duplication, conserved motifs and gene structures and phylogenetic relationships and cis-acting regulatory elements. Through the co-expression analysis on different transcriptomic data, five *MePMEs* were selected. A further expression pattern analysis of the shared *MePMEs* under different stresses and in different tissues suggested that *MePME1* may be the member of the PME family that is worthy of the deepest investigation. The findings from this research thus provide valuable information for further functional investigations on cassava *PME* genes in the near future.

## 4. Materials and Methods

### 4.1. Identification of Pectin Methylesterase Gene Family Members in Cassava

The protein family (PFAM) serial number of the PME domain (PF01095) was used as keyword to search the cassava genome database v6.1 (https://Phytozome-next.jgi.doe.gov/info/Mesculenta_v6_1, accessed on 18 April 2022) [67]. This allowed us to identify all cassava *MePME* members and download their annotation information. Next, we filtered the *MePME* information to find the genes with a PFAM serial number indicating the PMEI domain (PF04043), and these genes were classified as type-Ⅰ *MePMEs*. The residual genes without a PMEI domain were classified as type-Ⅱ *MePMEs*.

### 4.2. Characterization of MePMEs

The sequences of *MePME* proteins were next submitted to the ProtParam tool in the ExPASy web page (https://web.expasy.org/protparam/, accessed on 20 April 2022) [47] in order to generate the basic properties, which included the protein length (aa), molecular weight (MW), theoretical isoelectric point (pI), aliphatic index, instability index and grand average of hydropathicity. If the pI value is greater than 7, the *MePME* is classified as an alkaline protein, if the pI value is less than 7, the *MePME* is classified as an acidic protein and if the pI value is 7, then the *MePME* is classified as a neutral protein. The instability index is used to evaluate the stability of a protein. When the instability index is less than 40, the protein is regarded as stable, while when the value is greater than 40, the protein is regarded as unstable [68]. The box and whiskers diagrams were outputted via Graphpad Prism 8. The signal peptide of each *MePME* was predicted by submitting the amino acid sequences to SignalP 6.0 [69] (https://services.healthtech.dtu.dk/services/SignalP-6.0, accessed on 17 April 2023).

### 4.3. Chromosomal Location, Duplication, Gene Structure, Conserved Motifs and Cis-Acting Regulatory Element Analysis of MePMEs

The CDS sequences, specific location information of each *MePME* gene on chromosome and the whole chromosome information of cassava were acquired from the cassava genomic database (https://Phytozome-next.jgi.doe.gov/info/Mesculenta_v6_1, accessed on 7 May 2023). Then, TBtools was used to calculate gene collinearity and export the graph [70]. For conserved motif analysis of all *MePMEs*, MEME (http://meme-suit.org, accessed on 7 May 2023) was used [71]. Gene structure analysis was generated using Gene Structure Server Display Server online (http://gsds.cbi.pku.edu.cn, accessed on May 2023) [72]. The type-Ⅰ *MePMEs* conserved motifs between PME domain and PMEI domain, which were analyzed via multiple alignment using ClustalW. The promoter sequence, which is 2000 bp upstream of start codon (ATG) of each MePME, was download from cassava genome, and inputted into PlantCARE [73] (http://bioinformatics.psb.ugen.be/webtools/plantcare/html, accessed on 24 October 2022) to search for the CAREs. The main CARE localizations on the promoter was visualized using TBtools. The number of each kind of CARE was statistic and visualized using Microsoft Excel.

### 4.4. Multiple Sequence Alignment and Phylogenetic Analysis of MePMEs with PMEs in Arabidopsis

Sixty-six PME protein sequences of *Arabidopsis* were downloaded from *Arabidopsis thaliana* genome TAIR10 (https://phytozome-next.jgi.doe.gov/info/Athaliana_TAIR10, accessed on 8 August 2022) [74]. Then, all the amino acid sequences of 89 *MePMEs* and 66 PMEs from *Arabidopsis* were aligned using ClustalW in MEGA11 software [48] with the default parameter. After that, the aligned sequences were used to generate a phylogenetic tree using the statistical method of maximum likelihood and Jones–Taylor–Thornton model. At last, the visualization and annotation of the phylogenetic tree graphic was obtained using the online tool iTOL v6 (https://itol.embl.de, accessed on 18 April 2023) [49].

### 4.5. Multifunctional MePME Gene Screening and Analysis of Their Expression Patterns

For screening *MePME* genes with multiple functions, cassava transcriptome data under different stresses were used to find the *MePME* family genes. The intact tuberous roots of PPD-sensitive cassava SC8 and PPD-tolerant cassava RYG1 were stored for 0 and 21 days and sampled to perform transcriptome analysis [41]. Two biological replicates were used. Transcriptome data with values of transcription per kilobase per million mapping readings (FPKM) in each sample were used to screen and count the number and gene IDs of *MePME* genes. SC124 cassava tuberous roots were crosscut into 5 mm thick slices, and stored in dark condition of 28 °C and 60% humidity for 0, 6, 12 and 48 h. Samples were then collected for transcriptome sequencing. To filter differentially expressed genes (DEGs) the screening condition was set as fold change ≥ 2 and *p* value ≤ 0.05. Three biological replicates were used [50]. *MePME* genes that were significantly expressed in at least one comparison group were selected. The intact cassava tuberous roots were soaked with 100 μmol/L melatonin for 2 h, and those treated with water were used as control. After that, the cassavas were cut crosswise into 5 mm thick slices and stored at 28 °C with 60% humidity in the dark for 6, 12 and 48 h to perform the transcriptome analysis. The screening condition for DEGs was fold change ≥ 2 and *p* value ≤ 0.05. Three biological replicates were used [51]. *MePME* genes, which were significantly expressed in at least one stage of melatonin-delayed PPD, were selected. SC124 cassava stem cuttings were cultured in pots containing red soil and vermiculite (1:1 in volume ratio) and irrigated using Hoagland’s solution once a week for 2 months. The uniform seedlings were selected for different chilling stress treatments, including 24 °C for 10 days as control, T1 (24 °C for 5 days + 14 °C for 5 days) as chilling acclimation, T2 (14 °C for 5 days + 4 °C for 5 days) as chilling stress after chilling acclimation, and T3 (24 °C for 5 days + 4 °C for 5 days) as chilling shock. The folded leaves, fully expanded leaves and roots after different chilling treatments at 6 h, 24 h and 5 d were collected to extract total RNA. Then, equal amounts of RNA samples for each time point and each organ of each treatment were collected and pooled together to generate mRNA libraries and sequenced via RNA-Seq. Three biological replicates were used [52]. Transcriptome data with FPKM values in each sample were used to screen *MePME* genes in response to chilling stress. Two-month-old TM60444 cassava seedlings were treated with 7% PEG and 50 μmol/L ABA, separately [75]. After 48 h of treatment, mature leaves were collected to extract total RNA and perform RNA-Seq analysis (PRJNA658570). Three biological replicates were used. Transcriptome data with FPKM values in each sample were used to screen *MePME* genes in response to PEG or ABA stress. Subsequently, the Omicshare tools, a free online platform for data analysis (https://www.omicshare.com/tools/Home/Soft/seniorvenn, accessed on 13 June 2023), was used to output UpSet Venn diagram and to illustrate the shared *MePMEs* in different transcriptome data. For expression pattern analysis of shared *MePMEs* under different stresses, the FPKM values from the above transcriptome were selected to generate histogram.

For tissue-specific expression analysis, TME 204 cassava seedlings cultured in a greenhouse for 3 months were used to harvest different tissue samples, including leaf blade, petiole, stem, shoot apical meristem, fibrous roots, root apical meristem and storage roots. In addition, the 4-week-old OES and 3-week-old FEC tissues of TME204 were also sampled. Total RNA was isolated from the above different samples for RNA-Seq analysis, separately (PRJNA324539). With the exception of two biological replicates set in storage roots, three biological replicates were set in each of the remaining eight samples [76]. The FPKM values of shared *MePMEs* in nine cassava tissues were selected from the transcriptome data for subsequent analysis. All the histograms were outputted via Graphpad Prism 8. The significant differences were evaluated using ordinary one-way ANOVA analysis and Dunnett’s multiple comparisons test.

## Data Availability

The datasets generated during and/or analyzed during the current study are available from the corresponding author on reasonable request.

## Supplementary Materials

The following supporting information can be downloaded at: https://www.mdpi.com/article/10.3390/plants12132529/s1, Table S1: Physical and chemical information of *MePMEs* in cassava, Table S2: Information of *MePMEs* duplication pairs, Table S3: signal peptide prediction of *MePMEs*, Table S4: *MePMEs* in different transcriptome data.
